# Comparative effectiveness of tocilizumab vs standard care in patients with severe COVID-19-related pneumonia: a retrospective cohort study utilizing registry data as a synthetic control

**DOI:** 10.1186/s12879-023-08840-6

**Published:** 2023-12-04

**Authors:** Yukari Uemura, Ryoto Ozaki, Tomohiro Shinozaki, Hiroshi Ohtsu, Yousuke Shimizu, Kazuo Izumi, Sho Saito, Nobuaki Matsunaga, Norio Ohmagari

**Affiliations:** 1 Biostatistics Section, Department of Data Science, Center for Clinical Sciences, National Center for Global Health and Medicine, 1-21-1 Toyama, Shinjyuku-ku, Tokyo, 162-8655 Japan; 2 Biometrics Department, Clinical Development Division, Chugai Pharmaceutical CO., LTD, Tokyo, Japan; 3 Department of Information and Computer Technology, Faculty of Engineering, Tokyo University of Science, Tokyo, Japan; 4 Clinical Pharmacology and Regulatory Sciences, Juntendo University Graduate School of Medicine, Tokyo, Japan; 5 Center for Clinical Sciences, National Center for Global Health and Medicine, Tokyo, Japan; 6 Disease Control and Prevention Center, National Center for Global Health and Medicine, Tokyo, Japan

**Keywords:** Tocilizumab, COVID-19-related pneumonia, Synthetic control, COVID-19 Registry Japan

## Abstract

**Background:**

The severity of coronavirus disease 2019 (COVID-19) infections has led to the development of several therapeutic agents, with tocilizumab becoming increasingly used to treat patients with COVID-19-related pneumonia. This study compared the use of tocilizumab treatment with the standard of care (SOC) to determine its efficacy against severe COVID-19-related pneumonia in Japan.

**Methods:**

This retrospective cohort study was designed to evaluate the efficacy of tocilizumab in two different databases: the JA42434 single-arm study and COVID-19 Registry Japan (COVIREGI-JP), with a synthetic control group from the COVIREGI-JP cohort as a benchmark for the tocilizumab group. The study’s primary objective was to evaluate the efficacy of tocilizumab in treating severe COVID-19-related pneumonia compared to the SOC among patients included in the above two databases. The SOC group was extracted as the synthetic control group using exact matching and a propensity score matching in sequence per subject. As a secondary objective, the efficacy of tocilizumab compared to the SOC was evaluated exclusively among patients included in the COVIREGI-JP database. In each objective, the primary endpoint was defined as the time to discharge or the status of awaiting discharge.

**Results:**

For the primary endpoint, the hazard ratio (HR) of the tocilizumab group against the SOC group was 1.070 (95% confidence interval [CI]: 0.565–2.028). The median time from Study Day 1 to discharge or the state of awaiting discharge was 15 days in the tocilizumab group and 16 days in the SOC group. The HRs for the secondary endpoints, namely, time to improvement in the clinical state, time to clinical failure, and time to recovery, were 1.112 (95% CI: 0.596–2.075), 0.628 (95% CI: 0.202–1.953), and 1.019 (95% CI: 0.555–1.871), respectively. Similarly, the HR of the primary endpoint for the secondary objective was 0.846 (95% CI: 0.582–1.230).

**Conclusions:**

Tocilizumab did not demonstrate a positive effect on time to discharge or the state of awaiting discharge. Furthermore, no statistically significant differences in other clinical outcomes, such as time to improvement in the clinical state, time to clinical failure, and time to recovery, were observed among the groups.

**Supplementary Information:**

The online version contains supplementary material available at 10.1186/s12879-023-08840-6.

## Background

Many individuals suffering from coronavirus disease 2019 (COVID-19) infections require hospital care due to a respiratory illness. Moreover, progressive critical illness with hypoxic respiratory failure may require prolonged ventilatory support. Therapeutic agents are being developed rapidly in response to the global COVID-19 pandemic. Several studies have reported an association between interleukin (IL)-6 and COVID-19 severity, which implies that IL-6 likely is related with the cytokine storm or acute respiratory distress syndrome [1–5]. Tocilizumab, a recombinant humanized monoclonal antibody directed at both the soluble and membrane-bound IL-6 receptors, has been used as a therapeutic agent to treat severe COVID-19-related pneumonia [6, 7].

There have been several clinical trials evaluating the effects of tocilizumab. The RECOVERY study included 4,116 hospitalized adult patients with COVID-19 characterized by hypoxemia and systemic inflammation. This trial compared the efficacy and safety of tocilizumab and the standard of care (SOC). For patients hospitalized with severe COVID-19, treatment with tocilizumab reduced mortality, increased the probability of successful hospital discharge, and reduced the need for invasive mechanical ventilation [8]. Moreover, recent retrospective studies have suggested that tocilizumab may be associated with a lower risk of death or intubation in patients with severe COVID-19 pneumonia [9–11].

Owing to national differences in factors such as the number of patients with COVID-19 relative to the population and healthcare systems, there may exist national differences in COVID-19 outcomes, including mortality rates [12]. Indeed, the characteristics and outcomes of hospitalized patients in Japan differed from those reported in other countries [13, 14]. Accordingly, a phase-III clinical study of tocilizumab for treating severe COVID-19-related pneumonia (JA42434 study) was performed in Japan. This study aimed to evaluate the efficacy, safety, pharmacodynamics, and pharmacokinetics of tocilizumab administered concomitantly with the SOC. However, the number of patients infected with COVID-19 in Japan was limited at the intended commencement of the study, making it potentially challenging to conduct a randomized clinical trial. Therefore, it was planned as a multi-site single-arm study. Thus, a more detailed study evaluating the efficacy of tocilizumab was considered appropriate.

Accordingly, we conducted a retrospective cohort study to evaluate the efficacy of tocilizumab compared with the SOC using that from two different databases: the JA42434 single-arm study and COVID-19 Registry Japan (COVIREGI-JP), with a synthetic control group from the COVIREGI-JP cohort included as a benchmark for the tocilizumab group. COVIREGI-JP is the largest registry of COVID-19 hospitalized patients in Japan. It is a database of reported COVID-19 cases centered on the National Center for Global Health and Medicine [13, 14]. The patients registered have been diagnosed as having COVID-19 and hospitalized at medical institutions in Japan. As of October 24, 2022, 73,898 patients from 687 participating institutions have been registered in this database.

This study’s primary objective was to evaluate the efficacy of tocilizumab on SOC in the JA42434 population by comparing it to a synthetic control group from the SOC population of the COVIREGI-JP cohort with severe COVID-19 pneumonia. As a secondary objective, the efficacy of tocilizumab compared to SOC was evaluated exclusively among participants included in the COVIREGI-JP database. To overcome potential bias associated with utilizing a synthetic control, appropriate design and analytical methods were used, including an exact and a propensity score matching in sequence per subject [15].

## Methods

### Study design and approval

To evaluate the efficacy of tocilizumab and SOC treatments against severe COVID-19-related pneumonia, a retrospective cohort study was conducted using data from the JA42434 study and the COVIREGI-JP database. The definitions of each cohort and group are as follows:

- JA42434 study tocilizumab set: patients who were registered in the JA42434 study and to whom tocilizumab was administered.
- COVIREGI-JP cohort tocilizumab and SOC sets: among patients registered in COVIREGI-JP, the set of patients who received tocilizumab treatment (tocilizumab set), and those who did not (SOC set). We used data from patients who had all the major observation items as of November 28, 2020 (i.e., frozen data as of November 28, 2020).
- Tocilizumab group and SOC group: matched pairs extracted from the tocilizumab and SOC sets following the matching algorithm defined beforehand. The matching was conducted separately for primary and secondary objectives.

**Fig. 1:**
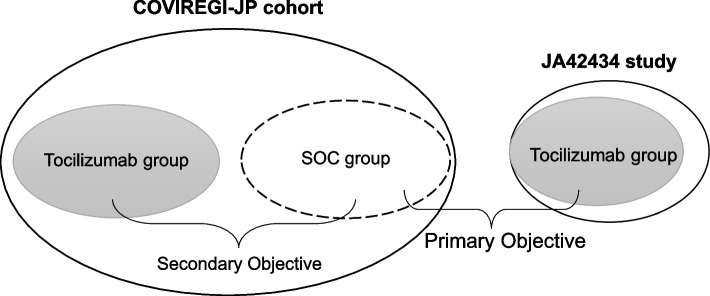
Framework for the primary and secondary objectives of this study. Primary objective: Compare the outcomes of the tocilizumab group from the JA42434 single-arm study with a synthetic control group from the COVIREGI-JP cohort. Secondary objective: Compare the outcomes of the tocilizumab and control groups exclusively among participants included in COVIREGI-JP

For the primary objective, primary and secondary endpoints were compared between the tocilizumab group extracted from the JA42434 study and the SOC group extracted from the COVIREGI-JP cohort SOC set. As a secondary objective, the endpoints were compared between the tocilizumab and SOC groups, each extracted from the COVIREGI-JP cohort tocilizumab and SOC sets (Fig. 1).

### Ethical approval

This study was conducted in accordance with ethical principles based on the Declaration of Helsinki and in compliance with the ethical guidelines for medical research involving human participants. It was approved by the National Center for Global Health and Medicine Ethics Review Committee (NCGM-G-004119-00) and registered in April 2020 in the UMIN Clinical Trials Registry (UMIN-CTR; ID: UMIN000043622). Informed consent from each patient was obtained in the form of opt-out and information regarding the opt-out approach of our study is available on the registry website. However, the participants of the source study, JA42434 study, provided informed consent.

### Study participants

The main inclusion criteria for the JA42434 study were as follows: patients who agreed to participate by signing a consent form, patients who were 18 years of age or older, patients hospitalized with COVID-19 pneumonia confirmed by a positive PCR test (respiratory, blood, urine, stool, or other bodily fluid samples) and chest X-radiography (X-ray) or computed tomography (CT) scan. In addition, the following pulmonary criteria were required: decreased oxygen saturation (SpO2 ≤ 93%) or hypoxemia (PaO2/FiO2 < 300 mmHg) under the SOC; otherwise, SpO2 ≤ 93% documented under lower supplemental oxygen or ambient air during the screening period, even if SpO2 > 93% under oxygen administration. Patients were excluded from the JA42434 study if they had a history of severe allergic reaction to tocilizumab or other monoclonal antibodies, were likely to die within 24 h regardless of treatment (in the opinion of the investigator or sub-investigator), were pregnant or lactating, or showed a positive pregnancy test before administration. In the JA42434 study, patients were enrolled from 12 enrollment sites, and the first-patient-in and last-patient-in dates were May 25, 2020 and October 31, 2020, respectively.

For the COVIREGI-JP cohort tocilizumab and SOC sets, we included patients who were at least 18 years old at the onset of COVID-19 and hospitalized between May 11 and November 28, 2020. We excluded pregnant patients and those who received sarilumab, baricitinib, or both drugs while hospitalized.

### Procedure

The observation items in this study were information collected in the JA42434 study and obtained from case report forms in the COVIREGI-JP database [13]. Study data were collected and managed by the Joint Center for Researchers, Associates and Clinicians (JCRAC) data center of the National Center for Global Health and Medicine using REDCap [16, 17].

### Endpoints, treatment strategies of interest, and follow-up

For the COVIREGI-JP cohorts, demographic information, epidemiological characteristics, comorbidities, signs, and symptoms were obtained during hospital admission (including conditions at admission). The outcome at discharge, as well as supportive care, history of drug administration, and complications during hospitalization were also collected. These data were collected on Days 1, 4, 8, 15, 22, and 29, starting from the date of admission [13].

In the JA42434 study, the following ordinal category scores were collected daily – 1: discharge from hospital or awaiting discharge; 2: oxygen administration unnecessary; 3: a cannula or mask, and reservoir, required for noninvasive oxygen administration; 4: bilevel positive airway pressure (BIPAP), continuous positive airway pressure (CPAP), or high flow required for noninvasive oxygen administration; 5: invasive mechanical ventilation required; 6: extracorporeal membrane oxygenation or invasive mechanical ventilation with additional organ support (renal replacement therapy, dialysis, cardiac stimulants, and/or vasoconstrictors) required; and 7: death [18]. For the COVIREGI-JP cohort tocilizumab and SOC sets, the clinical symptoms of each patient were classified into the above seven ordinal categories daily. This classification was conducted based on the data obtained during the study and the prespecified imputation algorithm (Supplementary Table 1; Additional file 1).

The primary endpoint was set as the time to discharge or the state of awaiting discharge (i.e., Category 1 on the seven-category ordinal scale). The secondary endpoints were as follows: (i) clinical state on the seven-category ordinal scale; (ii) time until improvement in clinical state on the seven-category ordinal scale; (iii) time until clinical failure (i.e., until the first occurrence of Category 5 or higher); (iv) time until recovery (i.e., until reaching Category 2); (v) mortality rate; (vi) proportion of patients using mechanical ventilators; and (vii) proportion of patients admitted to intensive-care units.

The follow-up time for each study was as follows: Each patient was observed until the earlier of the following: (i) Day 28 after the initial administration of the study drug (for the JP40959 study), hospitalization Day 29 from the date of admission (for the COVIREGI-JP cohort); (ii) death; and (iii) discharge or transfer from the hospital (only for the COVIREGI-JP cohort).

### Statistical methods

The COVIREGI-JP cohort SOC set was matched in variable ratios (1:1 to 4:1) to the JA42434 study tocilizumab set for the primary study objective and to the COVIREGI-JP cohort tocilizumab set for the secondary objective. Matching was performed using exact matching, followed by propensity score matching. To considerably minimize immortal time bias [19], the origin time (study Day 1) and evaluation period for time-to-event endpoints were defined as follows: First, we defined hospitalization Day 1 as the hospital admission date and then defined study Day 1 for the tocilizumab sets (in both JA42434 and COVIREGI-JP) as the initial tocilizumab administration date. Study Day 1 for patients in the SOC groups was defined according to their matched participant from the tocilizumab sets. Specifically, we sequentially conducted matching based on the information obtained during hospitalization Day 1 through study Day 1 for each patient in the tocilizumab sets. If SOC participants were matched to a tocilizumab participant, the hospitalization day on which the matched tocilizumab participant received treatment was taken to be study Day 1 for the SOC participants. For example, if matching was performed with a patient in the tocilizumab set for which tocilizumab was initially administered on hospitalization Day 5, then hospitalization Day 5 was taken to be study Day 1 for the matched patient in the COVIREGI-JP cohort SOC set and used as the origin baseline timepoint. For all patients in the tocilizumab and SOC groups, the evaluation period was set from study Day 1 to hospitalization Day 29.

Participants with either decreased oxygen saturation (SpO_2_ ≤ 93%) or hypoxemia (PaO_2_/FiO_2_ < 300 mmHg) from hospitalization Day 1 to study Day 1 were selected for each matching, ensuring maximum consistency with JA42434. We then selected SOC participants via exact matching based on the following variables measured between hospitalization Day 1 and study Day 1: abnormal pulmonary signs shown by thoracic X-ray or CT scan, the seven-category ordinal scale, invasive mechanical ventilation, and therapeutic drugs and methods (oral or intravenous steroids, antiviral agents [remdesivir and favipiravir], and plasmapheresis or plasma exchange). Among the patients selected, at most four SOC participants with the closest propensity scores within the prespecified caliper (0.2 times the standard deviation of the logit-transformed value for the propensity score in the tocilizumab group) were matched to each tocilizumab participant. The propensity score for each subject used in matching was estimated using a logistic regression model. Possible study Day 1 for the SOC set varied across matching-candidate patients in the tocilizumab sets. Hence, we used the following risk factors assessed on hospitalization Day 1 to estimate the propensity score of each patient: age, sex, weight, the use of invasive mechanical ventilation, comorbidities that are risk factors for increased severity, comorbidities that are risk factors for death, and comorbidities that are risk factors for both increased severity and death.

The participants in SOC groups were weighted using the reciprocal numbers of SOC participants in their matched sets (i.e., weights ranged from 0.25 to 1). For analysis of the primary endpoint, hospitalization Day 28 was handled as the censoring time if mortality events occurred during the evaluation period. The groups were compared using Kaplan–Meier plots and the Cox regression model. Nominal *P*-values were calculated using the Wald method. In addition, subgroup analysis for the primary endpoint was pre-planned for the use or non-use of remdesivir (between hospitalization Day 1 and study Day 1) and steroids (between hospitalization Day 1 and study Day 1), and the score on the seven-category ordinal scale on study Day 1 (Categories 2 to 3 and 4 to 5). Among secondary endpoints, time until recovery or improvement in the clinical state indicated by the seven-category ordinal scale was handled similarly. We also conducted a sensitivity analysis to adjust for censoring in post-hoc analyses. Given the number of censored cases in each objective, different analyses were conducted for primary and secondary objectives. For the primary outcome, the time to discharge or the state of awaiting discharge, transfer from the hospital was handled as censoring for the COVIREGI-JP cohort as hospital transfers prevented follow-up. We estimated the bounds by imputing the “extreme” outcome times, i.e., imputing “event occurrence” as soon as censored or “event-free” until the end of the follow-up for each censored case. Two pattern imputations for the censoring time, the best and worst scenarios for the tocilizumab group, were conducted. For the secondary objective, an inverse probability of censoring weighted (IPCW) method was conducted by adjusting for the baseline and time-varying variables listed below.

All statistical analyses were performed using SAS version 9.4 (SAS Institute, Cary, NC, USA).

## Results

### Flow diagram

**Fig. 2:**
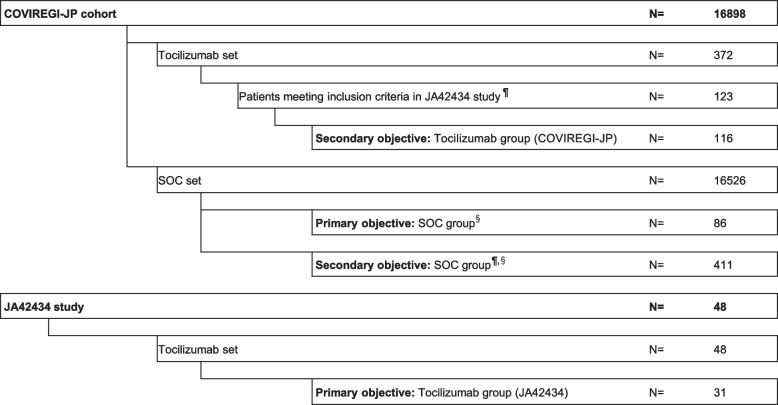
A flow diagram defining the analysis set for the study. ¶: Patients who meet the following JA42434 inclusion criteria: (i) evaluation performed between study Days –7 and 1; (ii) decreased oxygen saturation (SpO2 ≤ 93%) or hypoxemia (PaO2/FiO2 < 300 mmHg); and (iii) abnormal pulmonary signs shown by thoracic X-ray or CT scan. §: Same patient may be included in the SOC groups prepared for the primary and secondary objectives from the COVIREGI-JP cohort SOC set

The flow diagram in Fig. 2 shows the procedure up to defining the analysis sets. At the data extraction time for the COVIREGI-JP cohort, 16,898 patients were included, including 372 in the COVIREGI-JP cohort tocilizumab set, these being patients administered tocilizumab while hospitalized, and 16,526 in the COVIREGI-JP cohort SOC set, these being patients not administered tocilizumab. In addition, there were 48 patients in the JA42434 study tocilizumab set, these being patients administered tocilizumab.

For the primary objective analysis set, matching was performed with patients from the JA42434 study tocilizumab set and the COVIREGI-JP cohort SOC set. The tocilizumab and SOC groups included 31 and 86 patients, respectively. The mean matching ratio for each subject in this analysis set was approximately 2.77. Table 1 shows the patient demographic characteristics of the analysis set for the primary objective.

**Table 1:** Comparison of patient demographic characteristics of the analysis set for the primary objective

		Tocilizumab (JA42434)	SOC (COVIREGI-JP)	S.D.
		(*N* = 31.00)	(*N* = 31.00)	
Sex	Male	27.00 (87.1%)	26.75 (86.3%)	0.0
	Female	4.00 (12.9%)	4.25 (13.7%)	0.0
Age (yr)	Mean (SD)	66.3 (13.1)	67.7 (13.7)	–0.1
Age group (yr)	18–64	13.00 (41.9%)	12.00 (38.7%)	0.1
	65–84	14.00 (45.2%)	16.25 (52.4%)	–0.1
	≥85	4.00 (12.9%)	2.75 (8.9%)	0.1
Weight (kg)	Mean (SD)	70.6 (14.2)	71.4 (16.1)	0.0
Race	Japanese	29.00 (93.5%)	29.75 (96.0%)	–0.1
	Other (unknown)	2.00 (6.5%)	1.25 (4.0%)	0.1

*SOC* Standard treatment for patients with severe COVID-19; *S.D.* Standardized difference, *SD* Standard deviation

Weighted analysis was performed as appropriate to the matching ratio. For weighted analyses, numbers of participants are expressed to two decimal places.

The baseline characteristics for study Day 1 are provided in Table 2. In the analysis set for the primary objective, the proportion of men was approximately 86%, whereas the mean age was approximately 65 years. On study Day 1, approximately 70% of the participants had a score of 3 on the seven-category ordinal scale (i.e., a requirement other than for BIPAP/CPAP or high flow for noninvasive oxygen administration). In addition, the proportion of participants who used remdesivir was approximately 30%, and the median time after COVID-19 diagnosis was 4.0 days. It was confirmed that the groups had similar demographic characteristics, as well as previously administered and concomitant drugs.

**Table 2:** Comparison of patient baseline characteristics of analysis set for primary objective on study Day 1

			Tocilizumab (JA42434)	SOC (COVIREGI-JP)	S.D.
			(*N* = 31.00)	(*N* = 31.00)	
Smoking history		Current	6.00 (19.4%)	1.83 (5.9%)	0.4
		Former	12.00 (38.7%)	15.42 (49.7%)	–0.2
		Never	13.00 (41.9%)	9.25 (29.8%)	0.3
		Unknown	0.00 (0.0%)	4.50 (14.5%)	–0.6
Ordinal scale for clinical status		1	0.00 (0.0%)	0.00 (0.0%)	0.0
		2	3.00 (9.7%)	3.00 (9.7%)	0.0
		3	22.00 (71.0%)	22.00 (71.0%)	0.0
		4	2.00 (6.5%)	2.00 (6.5%)	0.0
		5	4.00 (12.9%)	4.00 (12.9%)	0.0
		6	0.00 (0.0%)	0.00 (0.0%)	0.0
		7	0.00 (0.0%)	0.00 (0.0%)	0.0
Mechanical ventilation		Yes	4.00 (12.9%)	4.00 (12.9%)	0.0
		No	27.00 (87.1%)	27.00 (87.1%)	0.0
Concomitant therapy on study Day 1	Steroid use	Yes	19.00 (61.3%)	22.67 (73.1%)	–0.3
		No	12.00 (38.7%)	8.33 (26.9%)	0.3
	Favipiravir use	Yes	9.00 (29.0%)	11.83 (38.2%)	–0.2
		No	22.00 (71.0%)	19.17 (61.8%)	0.2
	Remdesivir use	Yes	10.00 (32.3%)	10.00 (32.3%)	0.0
		No	21.00 (67.7%)	21.00 (67.7%)	0.0
	Plasmapheresis/plasma exchange	Yes	2.00 (6.5%)	0.00 (0.0%)	0.4
		No	29.00 (93.5%)	9.25 (29.8%)	1.7
Concomitant therapy on or after hospitalization Day 1 up to study Day 1	Steroid use	Yes	22.00 (71.0%)	23.67 (76.3%)	–0.1
		No	9.00 (29.0%)	7.33 (23.7%)	0.1
	Favipiravir use	Yes	9.00 (29.0%)	13.33 (43.0%)	–0.3
		No	22.00 (71.0%)	17.67 (57.0%)	0.3
	Remdesivir use	Yes	15.00 (48.4%)	12.00 (38.7%)	0.2
		No	16.00 (51.6%)	19.00 (61.3%)	-0.2
	Plasmapheresis/plasma exchange	Yes	2.00 (6.5%)	0.00 (0.0%)	0.4
		No	29.00 (93.5%)	31.00 (100.0%)	–0.4
Days from COVID-19 diagnosis		Mean (SD)	3.8 (2.0)	4.6 (2.7)	–0.4
Days from COVID-19 first hospitalization		Mean (SD)	4.5 (2.5)	4.5 (2.5)	0.0

*SOC* Standard treatment for patients with severe COVID-19, *S.D.* Standardized difference, *SD* Standard deviation

Weighted analysis was performed as appropriate to the matching ratio, and for weighted analyses, numbers of subjects are expressed to two decimal places

For the secondary objective analysis set, matching was performed using participant data from the COVIREGI-JP cohort tocilizumab and SOC sets. The tocilizumab and SOC groups included 116 and 411 subjects, respectively. The mean matching ratio in this analysis set was approximately 3.54. Patient demographic characteristics of the secondary objective analysis set are provided in Table 3.

**Table 3:** Comparison of patient demographic characteristics of the analysis set for secondary objective

		Tocilizumab (COVIREGI-JP)	SOC (COVIREGI-JP)	S.D.
		(*N* = 116.00)	(*N* = 116.00)	
Sex	Male	81.00 (69.8%)	83.50 (72.0%)	0.0
	Female	35.00 (30.2%)	32.50 (28.0%)	0.0
Age (yr)	Mean (SD)	69.1 (13.2)	69.5 (12.6)	0.0
Age group (yr)	18–64	41.00 (35.3%)	37.08 (32.0%)	0.1
	65–84	60.00 (51.7%)	66.67 (57.5%)	–0.1
	≥85	15.00 (12.9%)	12.25 (10.6%)	0.1
Weight (kg)	Mean (SD)	69.6 (17.1)	69.4 (17.5)	0.0
Race	Japanese	113.00 (97.4%)	112.25 (96.8%)	0.0
	Other (unknown)	3.00 (2.6%)	3.75 (3.2%)	0.0
BMI	<25	45.00 (38.8%)	47.33 (40.8%)	0.0
	≥25	50.00 (43.1%)	47.42 (40.9%)	0.0

*SOC* Standard treatment for severe COVID-19 patients, *S.D.* Standardized difference, *SD* Standard deviation

Weighted analysis was performed as appropriate to the matching ratio, and for weighted analyses, numbers of subjects are expressed to two decimal places.

Patient baseline characteristics of the analysis set for the secondary objective are provided in Table 4. The demographic characteristics were similar in the groups for the primary and secondary objectives.

**Table 4:** Comparison of patient baseline characteristics of analysis set for secondary objective at study Day 1

			Tocilizumab (COVIREGI-JP)	SOC (COVIREGI-JP)	S.D.
			(*N* = 116.00)	(*N* = 116.00)	
Smoking history		Current	10.00 (8.6%)	11.67 (10.1%)	0.0
		Former	37.00 (31.9%)	43.33 (37.4%)	–0.1
		Never	47.00 (40.5%)	38.75 (33.4%)	0.1
		Unknown	22.00 (19.0%)	21.42 (18.5%)	0.0
Ordinal scale for clinical status		1	0.00 (0.0%)	0.00 (0.0%)	0.0
		2	24.00 (20.7%)	24.00 (20.7%)	0.0
		3	59.00 (50.9%)	59.00 (50.9%)	0.0
		4	2.00 (1.7%)	2.00 (1.7%)	0.0
		5	31.00 (26.7%)	31.00 (26.7%)	0.0
		6	0.00 (0.0%)	0.00 (0.0%)	0.0
		7	0.00 (0.0%)	0.00 (0.0%)	0.0
Mechanical ventilation		Yes	31.00 (26.7%)	31.00 (26.7%)	0.0
		No	85.00 (73.3%)	85.00 (73.3%)	0.0
Concomitant therapy on study Day 1	Steroid use	Yes	88.00 (75.9%)	81.92 (70.6%)	0.1
		No	28.00 (24.1%)	34.08 (29.4%)	–0.1
	Favipiravir use	Yes	53.00 (45.7%)	33.25 (28.7%)	0.4
		No	63.00 (54.3%)	82.75 (71.3%)	–0.4
	Remdesivir use	Yes	64.00 (55.2%)	64.00 (55.2%)	0.0
		No	52.00 (44.8%)	52.00 (44.8%)	0.0
	Plasmapheresis/plasma exchange	Yes	0.00 (0.0%)	0.00 (0.0%)	0.0
		No	64.00 (55.2%)	57.83 (49.9%)	0.1
Concomitant therapy on or after hospitalization Day 1 up to study Day 1	Steroid Use	Yes	88.00 (75.9%)	84.67 (73.0%)	0.1
		No	28.00 (24.1%)	31.33 (27.0%)	–0.1
	Favipiravir use	Yes	61.00 (52.6%)	38.75 (33.4%)	0.4
		No	55.00 (47.4%)	77.25 (66.6%)	–0.4
	Remdesivir use	Yes	72.00 (62.1%)	67.50 (58.2%)	0.1
		No	44.00 (37.9%)	48.50 (41.8%)	–0.1
	Plasmapheresis / plasma exchange	Yes	0.00 (0.0%)	0.00 (0.0%)	0.0
		No	116.00 (100.0%)	116.00 (100.0%)	0.0
Days from COVID-19 diagnosis		Mean (SD)	4.7 (4.0)	4.0 (3.8)	0.2
Days from COVID-19 first hospitalization		Mean (SD)	3.4 (3.4)	3.4 (3.4)	0.0

SOC: Standard treatment for patients with severe COVID-19; S.D.: standardized difference; SD: standard deviation.

Weighted analyses were performed as appropriate to the matching ratio, and numbers of participants for weighted analyses are expressed to two decimal places

### Results for the primary endpoint

**Fig. 3:**
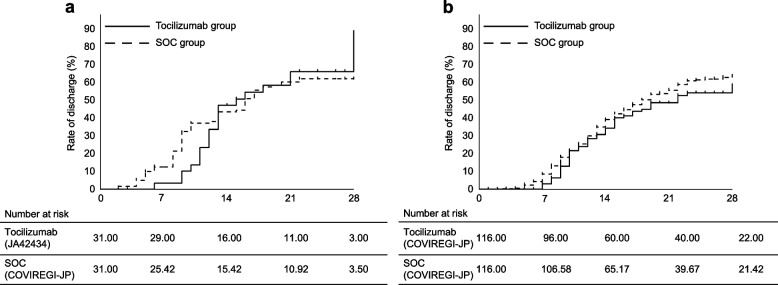
Kaplan–Meier curve for time to discharge or state of awaiting discharge. Panel **A**: Comparison between the tocilizumab and SOC groups for the primary objective. Panel **B**: Comparison between the tocilizumab and SOC groups for the secondary objective. Weighted analysis was performed as appropriate to the matching ratio, and the numbers of subjects are expressed in two decimal places for the weighted analyses

In the primary objective, the median time from study Day 1 to discharge or a state of awaiting discharge was 15 days in the JA42434 study tocilizumab set (95% confidence interval [CI]: 12–28 days) and 16 days in the SOC group (95% CI: 10 days to infinity). The hazard ratio (HR) for time until discharge from hospital or a state of awaiting discharge was 1.070 (95% CI: 0.565–2.028, *P* = 0.84). The Kaplan–Meier curves for time to discharge or a state of awaiting discharge are shown in Fig. 3A. The event occurrence rates by study Days 14 and 21, respectively, were 46.7% (95% CI: 30.9–65.7) and 65.5% (95% CI: 48.3–82.1) in the tocilizumab group and 43.1% (95% CI: 30.8–57.8) and 59.7% (95% CI: 46.5–73.3) in the SOC group (Table 5). The results of subgroup analysis were similar. The HRs obtained by sensitivity analysis for the censored cases were 1.315 (95% CI: 0.698–2.478, *P* = 0.40) and 0.845 (95% CI: 0.459–1.555, *P* = 0.59), respectively, for the best and worst scenarios for the tocilizumab group.

**Table 5:** Comparison between tocilizumab and SOC groups for time to discharge or state of awaiting discharge

		Comparison for primary objective		Comparison for secondary objective	
		Tocilizumab (JA42434)	SOC (COVIREGI-JP)	Tocilizumab (COVIREGI-JP)	SOC (COVIREGI-JP)
		*N* = 31.00	*N* = 31.00	*N* = 116.00	*N* = 116.00
HR		1.070 (0.565–2.028)	reference	0.846 (0.582–1.230)	reference
Event rate (%)	Study day 7	3.3	12.3	3.1	8.4
95% CI		(0.5–21.4)	(6.0–24.3)	(1.0–9.4)	(5.9–11.8)
	Study day 14	46.7	43.1	34.2	38.9
		(30.9–65.7)	(30.8–57.8)	(25.4–44.9)	(33.8–44.5)
	Study day 21	65.5	59.7	48.5	55.5
		(48.3–82.1)	(46.5–73.3)	(38.6–59.4)	(50.0–61.3)

*HR* Hazard ratio, *SOC* Standard treatment for patients with severe COVID-19; Event rate: event probability determined by the Kaplan–Meier method

Weighted analyses were performed as appropriate to the matching ratio, and numbers of participants for weighted analyses are expressed to two decimal places.

For the secondary objective, the median time from study Day 1 until discharge from hospital or a state of awaiting discharge was 22 days in the tocilizumab group (95% CI: 15 days to infinity) and 18 days in the SOC group (95% CI: 16–22 days). The inter-group HR of the tocilizumab group to the SOC group for time until discharge from hospital or a state of awaiting discharge was 0.846 (95% CI: 0.582–1.230, *P* = 0.38).The Kaplan–Meier curves for time until discharge from hospital or a state of awaiting discharge are shown in Fig. 3B. The HR adjusting for censoring through the IPCW method was estimated as 0.842 (95% CI: 0.623–1.138, *P* = 0.26). In addition, the event occurrence rates by study Days 14 and 21 were 34.2% (95% CI: 25.4–44.9) and 48.5% (95% CI: 38.6–59.4) and 38.9% (95% CI: 33.8–44.5) and 55.5% (95% CI: 50.0–61.3) in the tocilizumab and SOC groups, respectively (Table 5). Similar results were obtained during subgroup analysis.

### Results for secondary endpoints

The secondary endpoints were as follows: clinical symptoms according to the seven-category ordinal scale, time until improvement in clinical state, time until clinical failure, time until recovery, and mortality rate. No consistent trends between groups were found with these for either the primary or secondary objectives.

The distribution of clinical status using the seven-category ordinal scale for the primary objective is shown in Table 6. The median category scores for both the tocilizumab and SOC groups on study Days 14, 21, and 28 were 2.0, 1.0, and 1.0, respectively. The median time until improvement in clinical state was 13 days and 16 days for the tocilizumab and SOC groups, respectively (HR 1.112, 95% CI: 0.596–2.075, *P* = 0.74). The HRs for time to clinical failure or time to recovery were 0.628 (95% CI: 0.202–1.953, *P* = 0.42) and 1.019 (95% CI: 0.555–1.871, *P* = 0.95), respectively. Furthermore, the mortality rate in the tocilizumab and SOC groups was 9.7% and 19.6%, respectively. The estimated risk difference was –9.9% (95% CI: –28.0–9.4).

**Table 6:** Distribution of the clinical status for the primary objective on study Days 14, 21, 28

		Comparison for primary objective		Comparison for secondary objective	
		Tocilizumab (JA42434)	SOC (COVIREGI-JP)	Tocilizumab (COVIREGI-JP)	SOC (COVIREGI-JP)
		(*N* = 31.00)	(*N* = 31.00)	(*N* = 116.00)	(*N* = 116.00)
Study Day 14	n	30.00	27.67	89.00	102.67
	Median	2.00	2.00	3.00	2.00
	Difference in medians	0.00		1.00	
	95% CI	(–1.58, 1.58)		(0.21, 1.79)	
Study Day 21	n	28.00	27.67	88.00	98.58
	Median	1.00	1.00	2.00	1.00
	Difference in medians	0.00		1.00	
	95% CI	(0.00, 0.00)		(0.24, 1.76)	
Study Day 28	n	25.00	27.67	86.00	96.25
	Median	1.00	1.00	1.00	1.00
	Difference in medians	0.00		0.00	
	95% CI	(0.00, 0.00)		(0.00, 0.00)	

*SOC* Standard treatment for patients with severe COVID-19

Weighted analyses were performed as appropriate to the matching ratio, and numbers of participants for weighted analyses are expressed to two decimal places

The distribution of clinical status for the secondary objective is also shown in Table 6. The HRs for time until improvement in clinical state, time until clinical failure, and time until recovery were estimated as 0.844 (95% CI: 0.598–1.190, *P* = 0.33), 1.670 (95% CI: 0.981–2.844, *P* = 0.06), and 0.802 (95% CI: 0.567–1.136, *P* = 0.21), respectively. The risk difference for the mortality rate was 4.3% (95% CI: –5.2–13.8). Moreover, there were no differences between the groups.

## Discussion

This retrospective cohort study was designed to evaluate the efficacy of tocilizumab over the SOC for patients with severe COVID-19-related pneumonia in two different databases: the JA42434 single-arm study and the COVIREGI-JP data. A synthetic control group from the COVIREGI-JP cohort was utilized as a benchmark for the tocilizumab group. In this study, tocilizumab did not improve the time to discharge or the state of awaiting discharge. With respect to clinical symptoms based on the seven-category ordinal scale, time to improvement in clinical state, time to clinical failure, time to recovery, and mortality rate, no consistent improvement trend of tocilizumab was found for either the primary or secondary objectives.

Our study findings are inconsistent with those of previous trials that showed tocilizumab to be an effective treatment over the SOC for hospitalized patients with COVID-19 characterized by hypoxia [8, 20–22]. Results from the RECOVERY trial demonstrated that patients allocated to the tocilizumab group had a lower 28-day mortality rate than those who received standard medical care alone (621 [31%] of 2022 patients in the tocilizumab group vs 729 (35%) of 2094 patients in the SOC group; rate ratio 0.85; 95% CI, 0.76–0.94; *P* = 0.0028). Moreover, tocilizumab was associated with a greater probability of discharge from hospital within 28 days (57% vs 50%) [8]. The REMAP-CAP trial recruited critically ill patients within 24 h of organ support initiation and showed tocilizumab to be more effective than standard care in reducing the risk for continued organ support. This treatment protocol prolonged survival in critically ill patients within 24 h of starting organ support [22]. Furthermore, meta-analyses have demonstrated that tocilizumab reduced all-cause mortality by Day 28 compared with standard care alone [23, 24].

Our findings are consistent with those of another randomized clinical trial involving hospitalized adult patients with COVID-19 pneumonia and a Pao2/Fio2 ratio of 200–300 mmHg. Patients who received tocilizumab in the COVINTOC trial showed no benefit in terms of disease progression compared to those on standard care [25]. The COVINTOC trial reported that the primary endpoint, namely, the proportion of patients with progression of COVID-19 from moderate to severe or from severe to death up to Day 14, was not significantly different between the tocilizumab plus standard care and standard care alone groups. Similarly, the secondary endpoints that included time to clinical improvement according to COVID-19 grade and time to hospital discharge did not show any significant difference between the two groups [26]. Additionally, although a few retrospective studies in Japan have evaluated the efficacy of tocilizumab using real-world data, none of them demonstrated a significant efficacy of tocilizumab over the SOC [27, 28].

Here, the efficacy of tocilizumab compared to the SOC could not be demonstrated in terms of primary and secondary endpoints. Attempts to use real-world data as an external synthetic control for drug evaluation in single-arm clinical trials have received considerable interest in recent years [29–31]. We designed and analyzed the study based on the points discussed therein. However, there are several limitations with respect to the study results, and care is needed with result interpretation.

1. Limitations in the comparability of the two groups

The patients enrolled in the JA42434 study showed signs of pneumonia from study Days –2 to 1, with decreased oxygen saturation (SpO2 ≤ 93%) or hypoxemia (PaO2/FiO2 < 300 mmHg). Owing to difficulties with the stipulated evaluation time for the COVIREGI-JP cohort, patients who showed signs of pneumonia on study Days –7 to 1 were selected. Therefore, patients in the COVIREGI-JP cohort could have been selected without showing signs of pneumonia on study Days –2 to 1, which likely impacted the HR for the time to discharge or a state of awaiting discharge up to Days 7. For the tocilizumab group, study Day 1 was defined as the date of initial tocilizumab administration. In contrast, study Day 1 in the SOC group was defined using the hospitalization date of the matched tocilizumab participant. The hospitalization day of the matched tocilizumab patient was considered study Day 1 for the SOC participant. Although it would have been ideal to have included laboratory data on study Day 1 to match background factors, as the COVIREGI-JP cohort did not have sufficient laboratory data for all patients and the laboratory data collected after admission were limited, the study was not designed to include laboratory data for matching. Therefore, it is possible that confounding bias between the two groups of this study was not completely eliminated. To achieve more similar distributions of adjusted risk factors between the two groups, we narrowed the matching-caliper coefficient to 0.1. However, similar results were obtained for both the primary and secondary endpoints. The results of this sensitivity analysis were natural in the case where there were insufficient data to adjust risk factors.

2) Censored data, including subjects for whom follow-up was impossible

In the JA42434 study, progression could be followed up to Day 28, even after hospital transfers, except in patients who withdrew from the study. In the COVIREGI-JP cohort, hospital transfers could prevent follow-up of progression, which would affect evaluation. Thus, subjects for whom “the patient’s condition deteriorated and he/she was transferred to a different hospital” or “the patient’s condition improved and he/she was transferred to a different hospital” was noted may have led to selection bias. This may have led to the discrepancy in time to failure results between the primary and secondary objectives. Although we conducted sensitivity analyses using the bound approach for the primary analysis and the IPCW method for the secondary analysis, the interpretation of the results may be limited. As data on the conditions of patients immediately before censoring were not available, the selection bias may not have been adequately adjusted.

3) Matching

Although the target numbers of participants for the primary and secondary objectives in the SOC group were four times the respective numbers in the tocilizumab group, the number of subjects meeting the conditions for matching did not reach the expected number. Therefore, the number of participants included in the analysis was lower than the target number. Caliper matching using the propensity score and exact matching method was performed to increase the comparability between the two groups despite the smaller number of study participants included in the analysis.

## Conclusions

The effect of tocilizumab against COVID-19 pneumonia was evaluated in this retrospective cohort study. Our results indicated that tocilizumab treatment had no effect on outcomes, including on time to discharge. This study had several limitations, mostly due to the use of a synthetic control from the registry data.

### Supplementary Information

**Additional file 1: Supplementary Table 1.** The pre-specified imputation algorithm for the seven-category ordinal scale.

## Data Availability

The datasets generated and/or analyzed during the current study are not publicly available because of a contract with Chugai Pharmaceutical Co., but are available from the corresponding author upon reasonable request and with permission of the data owner.

## Abbreviations

CI: Confidence interval
COVIREGI-JP: COVID-19 Registry Japan
CT: Computed tomography
HR: Hazard ratio
SOC: Standard of care
X-ray: Chest X-radiography
