# Supplementary material for: Comparative effectiveness of tocilizumab vs standard care in patients with severe COVID-19-related pneumonia: a retrospective cohort study utilizing registry data as a synthetic control
Source: BMC Infect Dis. 2023 Dec 4;23:849. doi: 10.1186/s12879-023-08840-6 (PMC10694888; doi:10.1186/s12879-023-08840-6)
Supplement: Supplementary file 1 — Additional file 1: Supplementary Table 1. The pre-specified imputation algorithm for the seven-category ordinal scale. [file 12879_2023_8840_MOESM1_ESM.docx]

**Supplementary Table 1. The pre-specified imputation algorithm for the seven-category ordinal scale**

| Priority | Days during the visit at which data is collected | Imputed category |
| --- | --- | --- |
| 1 | - The period from the day after the last visit point evaluated as Category 6 or 5 to the day before the next time point. | Category 4 |
| 2 | - The period from the visit evaluated as Category 4 to the day before the seven-category ordinal scale is evaluated. | Category 4 |
| 3 | - The period from the visit evaluated as Category 3 to the day before the seven-category ordinal scale is evaluated. | Category 3 |
| 4 | - The period from the visit evaluated as Category 2 to the day before the seven-category ordinal scale is evaluated. | Category 2 |
| 5 | - The visit where the seven-category ordinal scale is not evaluated. | missing |

Researchers in the JA42434 study collected the following seven ordinal category scores daily, 1: discharge from hospital or awaiting discharge; 2: oxygen administration unnecessary; 3: a cannula or mask and reservoir required for noninvasive oxygen administration; 4: bilevel positive airway pressure, continuous positive airway pressure , or high flow required for noninvasive oxygen administration; 5: invasive mechanical ventilation required; 6: extracorporeal membrane oxygenation required, or invasive mechanical ventilation with additional organ support (renal replacement therapy, dialysis, cardiac stimulants, and/or vasoconstrictors) required; and 7: death. For the COVIREGI-JP cohort, most information is collected on Days 1, 4, 8, 15, 22, and 29 starting from the date of admission. Accordingly, the seven-category ordinal scale is imputed for the days not evaluated based on the pre-specified imputation algorithm shown above. To reduce bias due to differences in assessment frequency among the two studies, the categorical data evaluated on days besides hospital Day 1, 4, 8, 15, 22, and 29 were assessed as follows: Visit points evaluated as Category 1 and Categories 2 – 4 prior to the discharge date were handled as missing data and subsequently supplemented by the same algorithm as the table.
